# PNAB 2017 and the number of community health agents in primary care in Brazil

**DOI:** 10.11606/s1518-8787.2021055003005

**Published:** 2021-11-18

**Authors:** Deborah Ellen Wanderley Gomes Freire, Aldelany Ramalho Freire, Edson Hilan Gomes de Lucena, Yuri Wanderley Cavalcanti

**Affiliations:** I Universidade Federal da Paraíba Programa de Pós-Graduação em Odontologia Departamento de Clínica e Odontologia Social João Pessoa PB Brasil Universidade Federal da Paraíba. Departamento de Clínica e Odontologia Social. Programa de Pós-Graduação em Odontologia. João Pessoa, PB, Brasil

**Keywords:** Community Health Workers, supply & distribution, Healthcare Disparities, Program Evaluation, Primary Health Care

## Abstract

**OBJECTIVE:**

To analyze the effect of the 2017 Basic Primary Care Policy (PNAB) on the number of community health agents per primary health care team.

**METHODS:**

This is a cross-sectional, descriptive and analytical study using data available on the Ministry of Health platform called e-Gestor da Atenção Básica, about Brazil's 5,570 towns between October 2017 and December 2019. The survival of the number of towns that did not reduce the number of community health agents was analyzed according to region of the country, the Human Development Index (HDI), the Gini Inequality Index and population size. Cox regression was used to analyze the factors associated with a reduction in the number of CHAs after one month and, from then on, every three months until two years had passed since the publication of the 2017 PNAB Ordinance, considering p < 0.05.

**CONCLUSIONS:**

After two years, the greatest reduction was observed in towns in the Midwest and South regions, which presented a high HDI, lower inequality and larger populations. Towns in the Midwest (HR = 1.256) had a higher chance of reducing the number of CHAs compared to the North region. Towns with a higher HDI (HR = 1.053) and larger population size (HR = 1.186) were also more likely to reduc the number of community health agents. Therefore, after the 2017 PNA, the number of towns reducing the amount of community health workers in primary health care increased over the months

## INTRODUCTION

Primary Health Care (PHC) is considered to be initial point of contact of patients with the health system. It is also in charge of coordinating and structuring care^1,2^. In Brazil, PHC stands out as a comprehensive proposal for restructuring the health care system. The main milestone of this system was the implementation of the Family Health Program, subsequently known as the Family Health Strategy (FHS), after positive reviews of the Community Health Agents Program (PACS)^3^.

Community health (CHA) agents have played an important role in primary care^2,4^ after the program was restructured to decrease maternal and infant mortality through health promotion and disease prevention with home care^5,6^. They also create bonds with the community, promote humanization, patient reception and accountability^7^. Community agents also counsel the population on how to access and use health system services, carry out health surveillance actions, such as monitoring families in their assigned areas, in addition to developing activities to inform the population and prevent diseases^8,9^.

Typically, CHAs live in the community in which they operate, which strengthens the relationship of trust with users, playing a role in health surveillance and promotion^6^. Furthermore, being close to communities gives the agents a better understanding of the dynamics of the territory and the health needs of the communities and their users, which creates a bond between scientific knowledge and popular lore^6,7,10^.

Since the creation of the SUS, and especially after the expansion of the ESF, population health has advanced in many ways, such as the reduction of morbidity and mortality, especially infant and maternal; the reduction in the prevalence of malnutrition; decrease in preventable hospitalizations; increased immunization coverage; longer life expectancy at birth, improved access to water treatment, basic sanitation and health services resulting in greater equity, as well as increasing user satisfaction with health service care. All these achievements include the important role of CHAs.

However, the National Primary Care Policy (PNAB), published in October 2017 (Ordinance No. 2.436 / 2017 of the Ministry of Health), reduced the minimum amount of community agents in a ESF team from four to one, thus allowing a significant decrease of CHAs^8^, which can lead to reduced and poor population coverage.

The Ordinance also changed the role of CHAs in communities, allowing the inclusion of activities previously in the purview of endemic disease control agents (ACEs) into health surveillance, as well as operations performed especially by nursing technicians, such as blood pressure and capillary blood glucose monitoring and wound dressings. Such tasks, despite prioritizing the care pathways, impact the work of agents by hindering education, prevention and health promotion actions^2,12^.

As a result, the decreased coverage of CHAs can lead to the emergence of barriers to services and affect several processes established through the relationship of these workers with the population, particularly in the social determinants of the health-disease process^2,13^.

Due to its importance and potential impact on health, the objective of this study was to analyze the effect of the 2017 PNAB on the number of community agents per primary care team, between October 2017 and December 2019.

## METHODS

A cross-sectional, descriptive and analytical study was conducted using data from January 2017 to December 2019 about all 5,570 Brazilian towns published in public reports of the quantitative record of CHAs, available on the Ministry of Health platform e-Gestor da Atenção Básica do Ministério da Saúde^14^.

To analyze the factors associated with the reduction in the number of CHAs, the following independent variables were considered: Time: 1, 3, 6, 9, 12, 15, 18, 21 and 24 months, corresponding to the number of months passed since the publication of the new PNAB, in September 2017, Region: North, Northeast, Southeast, South, Midwest; the Human Development Index (HDI): Low (< 0.7), and High (≥ 0.7); the Gini Index: Less Unequal (≤ 0.61), and More Unequal (> 0,62); and Population size: up to 30 thousand inhabitants 30,001 of 50 thousand inhabitants, 50,001 to 100 thousand inhabitants, and more than 100 thousand inhabitants. The HDI and the Gini Index were obtained in the 2010 demographic Census, released by the United Nations Development Program (UNDP), which recorded 5,565 towns at the time. Population size was obtained in data from the Brazilian Institute of Geography and Statistics (IBGE). The dependent variable was “Variation in the number of CHAs”, categorized as: “did not change or increase” and “reduced” the number of CHAs in the town.

First, the data were analyzed descriptively to characterize the sample and obtain absolute and percentage distributions. Then, bivariate analysis was performed between the independent variables and the dependent variable. The Pearson chi-square test (χ^2^) was used to identify associations between the outcome variable and the independent variables.

Subsequently, multivariate analysis was performed to obtain the estimation of cumulative survival of Brazilian towns that did not reduce, that is, that kept or increased the number of community agents in teams. For this purpose, Cox regression was used to analyze the factors associated with the reduction in the number of CHAs after 1, 3, 6, 9, 12, 15, 18, 21 and 24 months since the new PNAB Ordinance was published. The information on Regions, HDI, Gini Index and population size were used as independent variables, which could predict the reduction in the number of CHAs, and were inserted into the model and underwent the backward stepwise (Wald) procedure. Variables with p > 0.20 were removed from the adjusted model and those with p < 0.05 were considered statistically significant. Risk ratio values (Harzard Ratio-HR) were obtained for each category of associated factors, considering the 95% confidence interval and the statistical significance of 5%. All tabulations and data analyses were performed in the statistical Package for Social Sciences software (IBM-SPSS, v. 24, IBM, Chicago, IL).

## RESULTS

**Figure 1:**
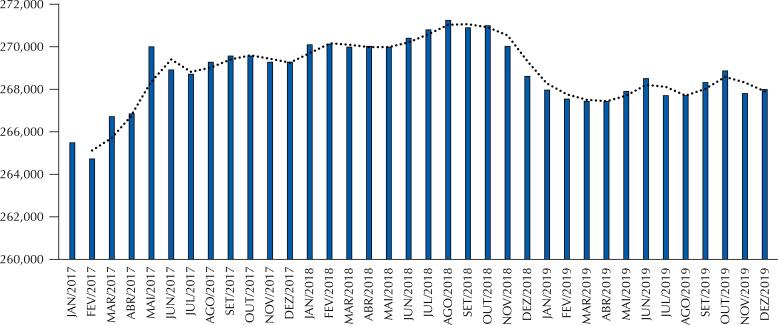
Number of teams of Community Health Agents (CHAs) in the Family Health Strategy (FHS) in Brazil, between January 2017 and December 2019.

The number of CHAs per family health team in Brazil, between October 2017 and December 2019, decreased from 269,577 to 268,879. The trend line shows the number of CHAs stabilizing only between February and May 2018, with the number varying upwards or downwards over the remaining months (Figure 1).

A significant reduction was found in the number of CHAs in Midwest (77.1%) and Southern (86.5%) towns, which showed a higher concentration of income (Gini index > 0.62) (72.3%) and population size up to 30 thousand inhabitants (86.9%). It was found that all variables exhibited statistical significance in the bivariate model, except the HDI (Table 1).

**Table 1:** Distribution of Brazilian towns regarding reduction in the number of Community Health Agents (CHAs) perm Family Health Team, according to the variables time since publication of the new PNAB, region of the country, Human Development Index (HDI), Gini Index and population size, between October 2017 and December 2019.

		Variation in the number of CHAs				p
		Did not reduce		Reduced		
		n	%	n	%	
Time	1 Month	5,090	91.4%	480	8.6%	< 0.001
	3 Months	4,693	84.3%	877	15.7%	
	6 Months	4,352	78.1%	1,218	21.9%	
	9 Months	4,203	75.5%	1,367	24.5%	
	12 Months	4,147	74.5%	1,423	25.5%	
	15 Months	3,883	69.7%	1,687	30.3%	
	18 Months	3,749	67.3%	1,821	32.7%	
	21 Months	1,603	28.8%	3,967	71.2%	
	24 Months	1,596	28.7%	3,970	71.3%	
Region	North	221	49.1%	229	50.9%	< 0.001
	Northeast	705	39.3%	1,089	60.7%	
	Midwest	107	22.9%	360	77.1%	
	Southeast	402	24.1%	1,266	75.9%	
	South	161	13.5%	1,030	86.5%	
HDI	Low HDI (< 0.7)	1,019	28.1%	2,612	71.9%	0.171
	High HDI (≥ 0.7)	576	29.8%	1,357	70.2%	
Gini Index	Less Unequal (≤ 0.61)	126	48.1%	136	51.9%	< 0.001
	More Unequal (> 0.62)	1,469	27.7%	3,834	72.3%	
Population size	Up to 30 thousand inhabitants	579	13.1%	3,828	86.9%	< 0.001
	30,001 to 50 thousand inhabitants	414	84.0%	79	16.0%	
	50,001 to 100 thousand inhabitants	306	87.7%	43	12.3%	
	More than 100 thousand inhabitants	297	93.7%	20	6.3%	

The region of the country, the HDI and population size were associated with the reduction in the number of CHAs per team in Brazil between October 2017 and September 2019. Towns in the Southeast (HR = 1.081), South (HR = 1.239) and Midwest (HR = 1.256) exhibited were more likely to reduce the number of teams compared to the North region. Towns with higher a HDI (HR = 1.053) and population size above 100 thousand inhabitants (HR = 1.186) were also more likely to reduce the number of CHAs per ESF team than towns with lower a HDI and population size up to 30 thousand inhabitants (Table 2).

**Table 2:** Cox regression to verify the factors associated with the reduction in the number of Community Health Agents (CHAs) per family health teams in Brazil after the publication of the new PNAB, between October 2017 and December 2019.

		B	Default Error	p	HR	Confidence Interval 95%	
						Bottom	Top
Region							
North					1.000		
	Northeast	0.000	0.032	0.990	1.000	0.939	1.064
	Midwest	0.228	0.039	< 0.001	1.256	1.164	1.356
	Southeast	0.078	0.034	0.020	1.081	1.012	1.154
	South	0.214	0.035	< 0.001	1.239	1.156	1.327
HDI							
	High HDI	0.052	0.020	0.011	1.053	1.012	1.096
	Low HDI				1.00		
Population Size							
Up to 30 thousand inhabitants					1.000		
	30,001 to 50 thousand inhabitants	-0.031	0.028	0.274	0.969	0.917	1.025
	50,001 to 100 thousand inhabitants	0.122	0.031	0.000	1.130	1.063	1.201
	> 100 thousand inhabitants	0.171	0.032	0.000	1.186	1.114	1.264

Note: Statistically significant: p < 0.05.

**Figure 2:**
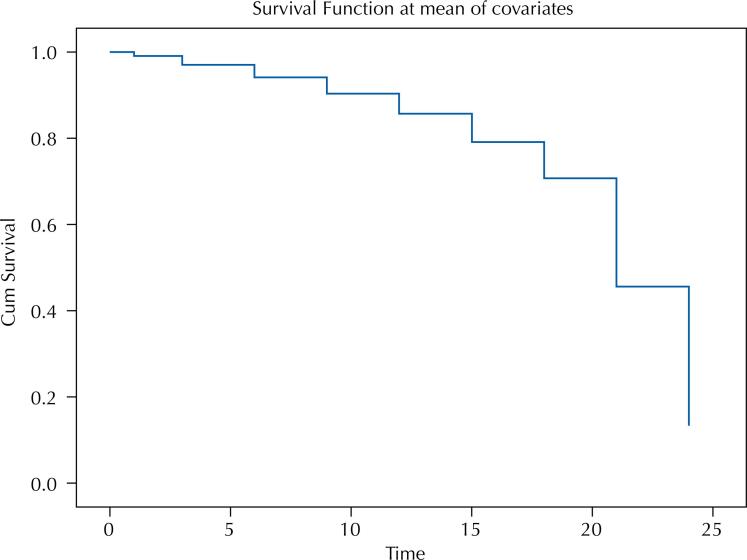
Cumulative survival of Brazilian towns that did not reduce the number of Community Health Agents (CHAs) after publication of the new PNAB Ordinance.

**Figure 3:**
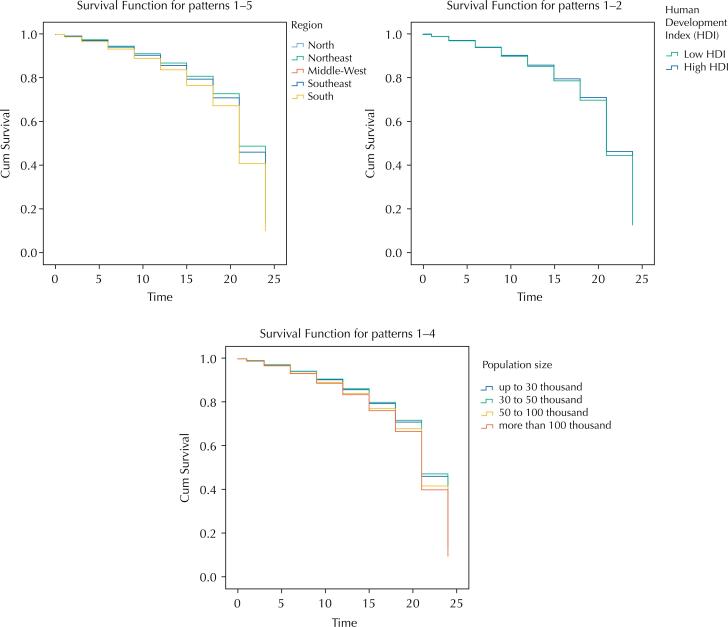
Cumulative survival of Brazilian towns that did not reduce the number of Community Health Agents (CHAs), between January 2017 and December 2019, according to the socioeconomic variables comprising the Cox regression model (a - Region, B - HDI, C - population size).

The survival curve of towns that did not reduce the number of CHAs decrease more significantly between September and December 2019 (Figure 2). Figure 3 illustrates the survival curves for each category of the variables under analysis. Towns in the Southern region showed a greater reduction between 20 and 24 months after the publication of the PNAB, followed by towns in the Midwest. The lowest survival rate was also found for towns with a higher HDI in the same period, as well as towns with a larger population size.

## DISCUSSION

The results of this study show a reduction in the number of CHAs per team in Brazil and an association between the increased chance of reducing this number and towns with a higher HDI, larger population size and located in the Southeast, South and Midwest regions.

Many countries have adopted the strategy of incorporating community health workers as a way of expanding coverage of services, particularly in areas where access is restricted. Brazil is a global example of successful PHC policy, thanks to the large-scale use of CHAs^15,16^. However, the results of this study show a significant reduction in the number of agents per FHS team in Brazilian towns.

The new PNAB caused a series of discontinuities for primary care; one of them is precisely the uncertainty regarding the number of CHAs per ESF team, dropping from a minimum of four agents to only one per team. In addition, CHAs are no longer required to cover 100% of the population assisted by the ESF program, only groups with greater vulnerability and risk, which are not properly defined by the Ordinance^2,8,12^.

Towns in the Southeast, South and Midwest regions were more likely to reduce the number of CHAs per team. The lower likelihood of reducing CHAs in the Northeast region may be associated with the history of the Community Health Agents Program (PACS), which was first conceived in Ceará State in 1987 and introduced health agents in the defunct Emergency Program with the aim of improving child health indicators. The repercussions of the work of agents led to their institutionalization in the creation of the Health Agents program. This successful experience inspired the nationwide implementation of the PACS, in 1991^15,17–19^.

Towns with a higher HDI underwent larger cuts in the number of community agents, which may imply a lesser demand for these workers in economically privileged towns since, as already mentioned, the CHA role was created to serve the poorest areas^20^ and is usually performed by a lay worker, usually with a low education and residing in the same community which they serve. Living in the community strengthens the bond between health teams and the families within the territory, the presence and work of CHAs improves relationships of trust and allow better identification of local problems^15,18^.

Towns with a population size above one hundred thousand inhabitants were also more likely to reduce CHAs when compared to smaller ones. The neglect of administrators toward health community agents is clear, especially in large cities, where they are regarded as expendable, unqualified and inefficient, an idea that favors health care focused on medical clinic to the detriment of whole care and the understanding of the health-disease process as an expression of social determinants in health. For this reason, CHAs were also delegated surveillance and nursing tasks, such as blood pressure and capillary glycemia measurement, demanding specific training from these workers so that they can carry on with their work^2,12,21^.

Another factor that may have contributed to the reduction of CHAs in large cities is: in 2014, the salary threshold of community agents was set and direct hiring became mandatory, which meant increased financial accountability for towns^22^ and less participation of States. In addition, underfunding of health is a historical problem in the country, and in 2016 Constitutional Amendment No 95 was passed^23^, freezing federal spending in a number of areas, including health, for 20 years, further increasing to the funding uncertainties of the sector^7,11^.

The importance of CHAs for PHC is highlighted in the literature^15,20^ after all, since the beginning of the PACS and the FHS, these workers have been responsible for many health promotion and disease prevention actions, both individually and collectively, allowing a better understanding of the social determinants of health, detection and monitoring of risk situations^13,15^. Moreover, they were also given the responsibility to extend the coverage of health services through low-complexity activities performed in home visits, but which had a huge impact on public health, particularly by decreasing maternal-infant mortality, such as oral rehydration therapy, increased vaccine coverage, encouragement of breastfeeding and maternal-infant follow-up^2,13,20^.

Home visits, still considered the most important activity of the CHA work routine, are a way of monitoring the health conditions of families and allow agents to identify situations that would not otherwise be noticeable. They are also an important way of listening to users and their demands. Community health agents play an important social role in promoting public policies aimed at improving their assigned territory, which have the potential to bring about positive health results in the most vulnerable communities^19,20,24^.

A systematic review on the effectiveness of CHAs has demonstrated positive effects related to maternal and child health, such as vaccination coverage, newborn and child mortality, and encouragement to exclusive breastfeeding, and weight control for children, besides positive effects on chronic non-communicable diseases, such as decreased hospitalization due to circulatory conditions and greater control of blood pressure by home monitoring performed by the agents^15^.

Thus, the reduction in the number of CHAs can have a negative impact on historical achievements of the PHC in the country, especially those related to patient care for chronic non-communicable diseases and maternal-child health. Since CHAs operate in areas that are more socially vulnerable, this reduction in the number of workers may hinder access of users under greater social risk to health services, in addition to interfering in the diagnosis of conditions determining the health-disease process.

The study used secondary data (socioeconomic and information) openly available in official databases. One must consider the possibility of inconsistency in the feeding of these databases, generating limitations specific to ecological studies. However, this study stands out in that it can be considered representative for Brazil, as the analysis includes all of the country's towns and provides a panorama consistent with the reality of the country. Studies like this are essential for monitoring health care and its impacts on the living and health conditions of Brazilians, with the permanent goal of improving and strengthening Brazil's Health System.

Therefore, this study showed that over the months, since the implementation of the 2017 PNAB, towns throughout the country have reduced their community agents in primary health care, especially towns in the Southeast, South and Midwest regions, which have a higher HDI, higher inequality rates and larger population sizes. The possible negative impact on the health achivements of PHC and population health indicators still need to be analyzed in subsequent studies.
