# Supplementary material for: PNAB 2017 and the number of community health agents in primary care in Brazil
Source: Rev Saude Publica. 2021 Nov 18;55:85. doi: 10.11606/s1518-8787.2021055003005 (PMC8659632; doi:10.11606/s1518-8787.2021055003005)
Supplement: Translation

# Translation: A PNAB 2017 e o número de agentes comunitários de saúde na atenção primária do Brasil

**Authors:** Deborah Ellen Wanderley Gomes Freire, Aldelany Ramalho Freire, Edson Hilan Gomes de Lucena, Yuri Wanderley Cavalcanti

**Affiliations:** I João Pessoa PB Brasil Universidade Federal da Paraíba. Departamento de Clínica e Odontologia Social. Programa de Pós-Graduação em Odontologia. João Pessoa, PB, Brasil

**Keywords:** Agentes Comunitários de Saúde, provisão & distribuição, Disparidades em Assistência à Saúde, Avaliação de Programas e Projetos de Saúde, Atenção Primária à Saúde

## Abstract

**OBJETIVO:**

Analisar o efeito da Política Nacional de Atenção Básica (PNAB) de 2017 no quantitativo de agentes comunitários de saúde, por equipe de atenção primária em saúde.

**MÉTODOS:**

Trata-se de um estudo transversal, descritivo e analítico, utilizando dados disponibilizados na plataforma e-Gestor da Atenção Básica, do Ministério da Saúde, sobre os 5.570 municípios brasileiros, entre outubro de 2017 e dezembro de 2019. A sobrevida do número de municípios que não reduziram o quantitativo de agentes comunitários de saúde foi analisada segundo a região do país, índice de desenvolvimento humano (IDH), índice de desigualdade de Gini e porte populacional. A Regressão de Cox foi utilizada para analisar os fatores associados à redução do número de ACS após um mês e, a partir de então, a cada três meses, até completarem os dois anos da publicação da portaria da PNAB 2017, considerando-se p < 0,05.

**CONCLUSÕES:**

Após dois anos, a maior redução foi observada em municípios das regiões Centro-Oeste e Sul, que apresentaram IDH alto, menos desiguais e com maior porte populacional. Municípios do Centro-Oeste (HR = 1,256) apresentaram maior chance de redução do número de ACS comparado à região Norte. Municípios com maior IDH (HR = 1,053) e maior porte populacional (HR = 1,186) também apresentaram maior chance de reduzir o número de agentes comunitários de saúde. Portanto, após a PNAB 2017, houve um aumento ao longo dos meses no número de municípios que reduziram a quantidade de agentes comunitários em saúde na atenção primária de saúde.

## INTRODUÇÃO

A Atenção Primária em Saúde (APS) é considerada o contato inicial do usuário com o sistema, além de ser responsável por coordenar e organizar o cuidado^1,2^. No Brasil, a APS se destaca por ser uma proposta mais abrangente de reorganização do modelo assistencial, cujo marco principal foi a implantação do Programa Saúde da Família, posteriormente reconhecido como Estratégia Saúde da Família (ESF), após avaliações positivas do Programa de Agentes Comunitários de Saúde (PACS)^3^.

Os agentes comunitários de saúde têm papel importante dentro da atenção primária^2,4^, com a reformulação do programa para reduzir a mortalidade materno-infantil por meio da promoção de saúde e prevenção de doenças com atendimento domiciliar^5,6^. Os ACS ainda atuam na criação de vínculos com a comunidade, promovendo humanização, acolhimento e responsabilização^7^. Os agentes comunitários ainda orientam a população sobre o uso e acesso aos serviços do sistema de saúde, realizam ações de vigilância em saúde, como acompanhamento das famílias em sua área de ação, além de desenvolver atividades de informação à população e de prevenção de doenças e agravos^8,9^.

Normalmente, os ACS residem na própria comunidade em que atuam, o que fortalece a relação de confiança com os usuários, exercendo seu papel na vigilância e promoção de saúde^6^. Além disso, sua proximidade com a comunidade confere ao agente melhor compreensão da dinâmica do território e as necessidades em saúde da comunidade e de seus usuários, construindo o elo entre o saber científico e o saber popular^6,7,10^.

Desde a criação do SUS e, principalmente, após a expansão da ESF, foram conquistados diversos avanços em saúde da população, como a redução da morbidade e mortalidade, especialmente infantil e materna; a redução na prevalência de subnutrição; diminuição de hospitalizações evitáveis; aumento da cobertura de imunização; maior expectativa de vida ao nascimento, melhorias no acesso ao tratamento de água, ao saneamento básico, e aos serviços de saúde, resultando em maior equidade, além de aumentar a satisfação do usuário com o atendimento do serviço de saúde. Todas essas conquistas tiveram a importante participação dos ACS.

No entanto, a Política Nacional de Atenção Básica (PNAB), publicada em outubro de 2017 (Portaria n° 2.436/2017 do Ministério da Saúde), reduziu o número mínimo de agentes comunitários em uma ESF, de quatro para um agente, permitindo significativa diminuição de ACS^8^, o que pode acarretar na redução e precarização da cobertura populacional.

A Portaria promoveu ainda descaracterização do papel do ACS junto às comunidades, possibilitando que se inclua atividades que eram inerentes aos agentes de combate a endemias (ACE) na vigilância em saúde, assim como operações desenvolvidas especialmente por técnicos em enfermagem, como aferição de pressão, glicemia capilar e curativos. Essas tarefas, apesar de priorizarem as linhas de cuidado, impactam no trabalho dos agentes, prejudicando a realização das ações de educação, prevenção e promoção em saúde^2,12^.

Dessa forma, a diminuição da cobertura dos ACS pode resultar no aparecimento de barreiras de acesso aos serviços e comprometer diversos processos já estabelecidos por meio da relação desse trabalhador com a população, especialmente nos determinantes sociais do processo saúde-doença^2,13^.

Por sua importância e potencial de impacto sobre a saúde, o objetivo deste estudo foi analisar o efeito da PNAB 2017 sobre o quantitativo de agentes comunitários por equipes de atenção primária, entre outubro de 2017 e dezembro de 2019.

## MÉTODOS

Foi realizado estudo transversal, descritivo e analítico, utilizando os dados disponibilizados nos relatórios públicos do histórico quantitativo de ACS, disponível na plataforma e-Gestor da Atenção Básica do Ministério da Saúde, no período de janeiro de 2017 a dezembro de 2019, de todos os 5.570 municípios brasileiros^14^.

Para análise dos fatores associados à redução do número de ACS, considerou-se como variáveis independentes: Tempo: 1, 3, 6, 9, 12, 15, 18, 21 e 24 meses, correspondente à quantidade de meses passados após a publicação da nova PNAB, em setembro de 2017; Região: Norte, Nordeste, Sudeste, Sul, Centro-Oeste; Índice de Desenvolvimento Humano (IDH): Baixo (< 0,7) e Alto (≥ 0,7); Índice de Gini: Menos Desigual (≤ 0,61) e Mais Desigual (> 0,62) e; Porte Populacional: até 30 mil habitantes, 30.001 a 50 mil habitantes, 50.001 a 100 mil habitantes e mais de 100 mil habitantes. O IDH e o Índice de Gini foram obtidos pelo Censo Demográfico de 2010, divulgado pelo Programa das Nações Unidas para o Desenvolvimento (PNUD), que contabilizava 5.565 municípios à época, e o Porte Populacional, obtido dos dados do Instituto Brasileiro de Geografia e Estatística (IBGE). A variável dependente foi “Variação no número de ACS”, categorizada em: “Não alterou ou aumentou” e “reduziu” o número de ACS do município.

Inicialmente os dados foram analisados descritivamente para caracterizar a amostra, obtendo as distribuições absolutas e percentuais. Em seguida, foi realizada análise bivariada entre as variáveis independentes e a variável dependente. O teste qui-quadrado de Pearson (χ^2^) foi utilizado para identificar associações entre a variável desfecho e as variáveis independentes.

Posteriormente, foi realizada análise multivariada, para se obter o cálculo de sobrevida acumulado dos municípios brasileiros que não reduziram, isto é, que mantiveram ou aumentaram o número de agentes comunitários nas equipes. Para tanto, utilizou-se a Regressão de Cox, analisando os fatores associados à redução do número de ACS após 1, 3, 6, 9, 12, 15, 18, 21 e 24 meses da publicação da portaria da nova PNAB. As informações sobre Região, IDH, Índice de Gini e Porte Populacional foram utilizadas como variáveis independentes, que poderiam predizer a redução do número de ACS, e foram inseridas no modelo e submetidas ao ajuste por meio do procedimento *stepwise backward: Wald*. Variáveis com p > 0.20 foram removidas do modelo ajustado e aquelas com p < 0.05 foram consideradas estatisticamente significantes. Valores de razão de risco (Harzard Ratio – HR) foram obtidos para cada categoria dos fatores associados, considerando-se o intervalo de confiança de 95% e a significância estatística de 5%. Todas as tabulações e análises dos dados foram realizadas no *software Statistical Package for Social Sciences* (IBM-SPSS, v.24, IBM, Chicago, IL).

## RESULTADOS

O número de ACS por Equipes de Saúde da Família no Brasil, entre outubro de 2017 e dezembro de 2019, reduziu de 269.577 para 268.879. A linha de tendência indica uma estabilização no quantitativo desses profissionais apenas entre fevereiro e maio de 2018, variando entre aumento e redução nesse número ao longo dos demais meses (Figura 1).

**Figura 1:** Número de Equipes de Agentes Comunitários em Saúde (ACS) na Estratégia Saúde da Família (ESF) no Brasil, entre janeiro de 2017 e dezembro de 2019.

Observou-se que houve significativa redução no número de ACS em municípios das regiões Centro-Oeste (77,1%) e Sul (86,5%), que apresentam maior concentração de renda (índice de Gini > 0,62) (72,3%) e com porte populacional até 30 mil habitantes (86,9%). Observou-se que todas as variáveis apresentaram significância estatística no modelo bivariado, exceto o IDH (Tabela 1).

**Tabela 1:** Distribuição dos municípios brasileiros em relação à redução no número de Agentes Comunitários de Saúde (ACS) por Equipes de Saúde da Família, segundo as variáveis tempo desde publicação da nova PNAB, Região do país, Índice de Desenvolvimento Humano (IDH), Índice de Gini e Porte Populacional, entre outubro de 2017 e dezembro de 2019.

		Variação no número de ACS				p
		Não reduziu		Reduziu		
		n	%	n	%	
Tempo	1 Mês	5.090	91,4%	480	8,6%	< 0,001
	3 Meses	4.693	84,3%	877	15,7%	
	6 Meses	4.352	78,1%	1.218	21,9%	
	9 Meses	4.203	75,5%	1.367	24,5%	
	12 Meses	4.147	74,5%	1.423	25,5%	
	15 Meses	3.883	69,7%	1.687	30,3%	
	18 meses	3.749	67,3%	1.821	32,7%	
	21 meses	1.603	28,8%	3.967	71,2%	
	24 meses	1.596	28,7%	3.970	71,3%	
Região	Norte	221	49,1%	229	50,9%	< 0,001
	Nordeste	705	39,3%	1.089	60,7%	
	Centro-Oeste	107	22,9%	360	77,1%	
	Sudeste	402	24,1%	1.266	75,9%	
	Sul	161	13,5%	1.030	86,5%	
IDH	IDH Baixo (< 0,7)	1.019	28,1%	2.612	71,9%	0,171
	IDH Alto (≥ 0,7)	576	29,8%	1.357	70,2%	
Índice de Gini	Menos Desigual (≤ 0,61)	126	48,1%	136	51,9%	< 0,001
	Mais Desigual (> 0,62)	1.469	27,7%	3.834	72,3%	
Porte Populacional	até 30 mil habitantes	579	13,1%	3.828	86,9%	< 0,001
	30.001 a 50 mil habitantes	414	84,0%	79	16,0%	
	50.001 a 100 mil habitantes	306	87,7%	43	12,3%	
	Mais de 100 mil habitantes	297	93,7%	20	6,3%	

A região do país, o IDH e o porte populacional foram associados à redução do número de ACS por equipes no Brasil entre outubro de 2017 e setembro de 2019. Municípios das regiões Sudeste (HR = 1,081), Sul (HR = 1,239) e Centro-Oeste (HR = 1,256) apresentaram maior chance de redução do número de equipes comparadas à região Norte. Municípios com maior IDH (HR = 1,053) e com porte populacional acima de 100 mil habitantes (HR = 1,186) também apresentaram maior chance de reduzir o número de ACS por ESF do que municípios com menor IDH e porte populacional de até 30 mil habitantes (Tabela 2).

**Tabela 2:** Regressão de Cox para verificar os fatores associados à redução do número de Agentes Comunitários de Saúde (ACS) por Equipes de Saúde da Família no Brasil, após a publicação da nova PNAB, entre outubro de 2017 e dezembro de 2019.

		B	Erro Padrão	p	HR	Intervalo de Confiança 95%	
						Inferior	Superior
Região							
	Norte				1,000		
	Nordeste	0,000	0,032	0,990	1,000	0,939	1,064
	Centro-Oeste	0,228	0,039	< 0,001	1,256	1,164	1,356
	Sudeste	0,078	0,034	0,020	1,081	1,012	1,154
	Sul	0,214	0,035	< 0,001	1,239	1,156	1,327
IDH							
	IDH Alto	0,052	0,020	0,011	1,053	1,012	1,096
	IDH baixo				1,00		
Porte Populacional							
	Até 30 mil habitantes				1,000		
	30 a 50 mil habitantes	-0,031	0,028	0,274	0,969	0,917	1,025
	50 a 100 mil habitantes	0,122	0,031	0,000	1,130	1,063	1,201
	> 100 mil habitantes	0,171	0,032	0,000	1,186	1,114	1,264

Nota: Estatisticamente significante (p < 0,05)

A curva de sobrevida dos municípios que não reduziram o número de ACS demonstrou maior queda entre setembro e dezembro de 2019 (Figura 2). A Figura 3 ilustra as curvas de sobrevida para cada categoria das variáveis em análise. Municípios da região Sul apresentaram maior redução entre 20 e 24 meses após a publicação da PNAB, seguidos pelos municípios da região Centro-Oeste. A menor taxa de sobrevida também foi observada para municípios com maior IDH no mesmo período, bem como municípios com maior porte populacional.

**Figura 2:** Sobrevida acumulada dos municípios brasileiros que não reduziram o número de Agentes Comunitário de Saúde (ACS) após publicação da Portaria da nova PNAB.

**Figura 3:** Sobrevida acumulada dos municípios brasileiros que não reduziram o número de Agentes Comunitários de Saúde (ACS), entre janeiro de 2017 e dezembro de 2019, segundo as variáveis socioeconômicas que compuseram o modelo de regressão de Cox (A - Região, B - IDH, C - Porte Populacional).

## DISCUSSÃO

Os resultados deste estudo apontam para uma redução no número de ACS por equipes no Brasil e uma associação entre o aumento da chance de reduzir esse número e municípios com maior IDH, maior porte populacional e localizados nas regiões Sudeste, Sul e Centro-Oeste.

Muitos países têm adotado a estratégia de incorporar profissionais de saúde comunitários como forma de ampliar a cobertura dos serviços, principalmente em áreas onde o acesso é limitado. O Brasil é exemplo mundial de política bem-sucedida na APS, graças ao uso em larga escala de ACS^15,16^. Porém, os resultados deste estudo apontam para uma redução significativa no número de agentes por ESF nos municípios brasileiros.

A nova PNAB provocou uma série de descontinuidades para a atenção primária, uma delas está justamente na indefinição do número de ACS por ESF, passando de um mínimo de quatro agentes para apenas um por equipe. Além disso, não é mais necessário que haja 100% de cobertura da população da ESF por parte do agente comunitário, apenas de grupos de maior vulnerabilidade e risco, que não são devidamente definidos seus parâmetros pela Portaria^2,8,12^.

Os municípios das regiões Sudeste, Sul e Centro-Oeste tiveram mais chance de reduzir o número de ACS por equipe. A menor chance de redução de ACS na região Nordeste pode estar associada ao histórico do PACS, que teve seu embrião no Ceará, em 1987, uma iniciativa que inseria agentes de saúde no extinto Programa de Emergência com objetivo de melhorar os indicadores de saúde infantil. A repercussão do trabalho das agentes acarretou sua institucionalização por meio da criação do Programa Agentes de Saúde. Essa experiência exitosa inspirou a implantação nacional do PACS, em 1991^15,17–19^.

Municípios com maior IDH sofreram maior redução no número de agentes comunitários, o que pode significar desvalorização do papel desse profissional em municípios socioeconomicamente privilegiados, pois, como já citado, o posto de ACS foi criado para atender às áreas mais pobres^20^ e representava um trabalhador leigo, geralmente de baixa escolaridade e morador da própria comunidade. O fato de residirem na comunidade fortalece o elo entre as equipes de saúde e as famílias do território, a presença e atuação do ACS favorece as relações de confiança que permitem melhor identificação dos problemas locais^15,18^.

Municípios com porte populacional acima de cem mil habitantes também tiveram maior chance de redução de ACS quando comparados aos de menor porte. É visível o descaso de gestores, especialmente em municípios de grande porte, com o trabalho dos agentes comunitários, vendo-os como prescindíveis, de baixa complexidade e baixa resolutividade, ideia que privilegia o cuidado pautado na clínica médica, em detrimento do cuidado integral e compreensão do processo saúde-doença como expressão dos determinantes sociais em Saúde, por isso também foram atribuídas ao ACS atividades em vigilância e de enfermagem, como aferição de pressão arterial, glicemia capilar, exigindo desses profissionais uma formação específica para que possam continuar seu atuando^2,12,21^.

Outro fator que pode ter contribuído para redução de ACS em municípios de grande porte: em 2014, definiu-se o piso salarial dos agentes comunitários e estabeleceu-se a obrigatoriedade de contratação por vínculo direto, isso significou aumento na responsabilização financeira dos municípios^22^, diminuindo a participação do poder público estadual. Ademais, o subfinanciamento do setor de saúde é um problema histórico no país e, em 2016, foi aprovada a Emenda Constitucional n° 95^23^, que congelou os gastos públicos federais em diversas áreas, incluindo a Saúde, por 20 anos, o que aumenta ainda mais as incertezas de financiamento do setor^7,11^.

A importância do ACS para a APS é destacada na literatura^15,20^, afinal, desde o início do PACS e da ESF, esses profissionais foram responsáveis por muitas ações de promoção da saúde e prevenção de doenças, individuais e coletivas, possibilitando melhor compreensão dos determinantes sociais em saúde, da detecção e do monitoramento de situações de risco^13,15^. Além disso, também lhes foi conferida a responsabilidade de estender a cobertura dos serviços de saúde, por meio de atividades de baixa complexidade realizadas durantes as visitas domiciliares, mas que tiveram enorme impacto na saúde pública, principalmente na redução da mortalidade materno-infantil, como a terapia de reidratação oral, aumento da cobertura vacinal, estímulo ao aleitamento materno e acompanhamento materno-infantil^2,13,20^.

A visita domiciliar, considerada ainda a atividade mais importante da rotina de trabalho do ACS, permite que as condições de saúde das famílias sejam constantemente monitoradas, possibilitando a identificação de situações que, de outra forma, não se manifestariam, além de ser uma importante ação de acolhimento aos usuários e suas demandas. O ACS tem importante papel social, favorecendo a promoção de políticas públicas voltadas para melhorias em seu território de adscrição, que podem ter potencial para obter resultados positivos na saúde das comunidades mais vulneráveis^19,20,24^.

Uma revisão sistemática sobre a efetividade dos ACS demonstrou efeitos positivos relacionados à saúde materno-infantil, como cobertura vacinal, mortalidade infantil e neonatal, estímulo à amamentação exclusiva e controle de peso de crianças, além de efeitos positivos sobre doenças crônicas não transmissíveis, a exemplo da redução da hospitalização por condições circulatórias e maior controle da hipertensão arterial pelo monitoramento domiciliar realizado por estes profissionais^15^.

Assim, a redução no número de ACS pode impactar negativamente conquistas históricas obtidas com a APS no país, especialmente as relacionadas ao cuidado em pacientes com doenças crônicas não transmissíveis e à saúde materno-infantil, uma vez que os ACS atuam em áreas de maior vulnerabilidade social, essa redução no quadro de profissionais pode dificultar o acesso dos usuários de maior risco social aos serviços de saúde, além de interferir no diagnóstico de condições determinantes do processo saúde-doença.

O estudo utilizou dados secundários (socioeconômicos e informações) disponibilizados abertamente em bases de dados oficiais. Deve-se considerar a possibilidade de inconsistências na alimentação dessas bases, gerando limitações próprias de estudos ecológicos. Entretanto, este estudo destaca-se por poder ser considerado representativo para o Brasil, considerando para análise todos os seus municípios e fornecendo um panorama compatível com a realidade nacional. Estudos como esse são essenciais para o monitoramento da atenção à saúde, bem como de seus impactos nas condições de vida e saúde dos brasileiros, visando sempre à qualificação e ao fortalecimento do Sistema Único de Saúde.

Portanto, o presente estudo demonstrou que ao longo dos meses, desde a implantação da PNAB 2017, municípios de todo o país reduziram seus quadros de agentes comunitários na atenção primária em Saúde, especialmente aqueles das regiões Sudeste, Sul e Centro-oeste, que apresentam um maior IDH, apresentam índices de maior desigualdade e de maior porte populacional. O possível impacto negativo sobre as conquistas em saúde da APS e os indicadores de saúde da população ainda precisam ser analisados em estudos posteriores.
